# ANKRD26 Gene Variant of Uncertain Significance in a Patient With Acute Myeloid Leukemia

**DOI:** 10.7759/cureus.36152

**Published:** 2023-03-14

**Authors:** Benjamin J McCormick, Razvan M Chirila

**Affiliations:** 1 Internal Medicine, Mayo Clinic, Jacksonville, USA

**Keywords:** ankrd26, thrombocytopenia, variant of uncertain significance, acute myeloid leukemia (aml), chat gpt

## Abstract

*ANKRD26*-related thrombocytopenia is a rare inherited disorder associated with an increased risk of malignancy. While the genetic mutations underlying this condition are well understood, there is limited knowledge regarding its contribution to myeloid neoplasms, such as acute myeloid leukemia (AML). We present a case of *ANKRD26*-related thrombocytopenia with a variant of uncertain significance in a patient with AML and review the pathogenesis and implications of hereditary germline mutations in disease management.

## Introduction

*ANKRD26*-related thrombocytopenia is a rare genetic disorder that affects platelet production and is associated with an increased risk of developing acute myeloid leukemia (AML). The *ANKRD26* gene encodes proteins containing N-terminal ankyrin repeats, which regulates proteins in mitogen-activated protein kinase (MAPK) signaling pathways involved in megakaryocyte maturation and platelet formation [1]. Pathogenic variants reported in *ANKRD26* are found in the 5' untranslated region, result in a gain-of-function effect, and cause *ANKRD26*-related thrombocytopenia, also known as thrombocytopenia-2 [2]. Increased risks for myeloid malignancies include acute leukemias, myelodysplastic syndromes, and chronic myeloid leukemia, which is thought to be secondary to gain-of-function mutations with pathogenic overexpression of *ANKRD26* [3]. Penetrance of thrombocytopenia appears to be near complete; however, interfamilial and intrafamilial variability have been described regarding myeloid malignancies [2-4].

## Case presentation

A 20-year-old female with past medical history of childhood Diamond-Blackfan anemia in remission presented to our hospital for initiation of induction chemotherapy for high-risk AML without maturation with complex cytogenetics, including *p53* mutation, that was diagnosed following a workup for menorrhagia. At the time of admission, complete blood count revealed white blood cell count 2.6x10^9^/L (normal range, 3.4-9.6x10^9^/L), hemoglobin 10.2 g/dL (normal range, 11.6-15.0 g/dL), and platelet count 11x10^9^/L (normal range, 157-371x10^9^/L). During evaluation for potential hematopoietic stem cell transplantation genomic analysis was performed with pertinent results shown in Table 1.

**Table 1:** Next-generation sequencing genomic report from GeneDx demonstrating ANKRD26 variant of uncertain significance

Gene	Disease	Mode of Inheritance	Variant	Zygosity	Inherited Form	Classification
ANKRD26	*ANKRD26*-related thrombocytopenia and myeloproliferative neoplasms	Autosomal Dominant	c.5106 G>C p.(Q1702H)	Heterozygous	Unknown	Variant of Uncertain Significance

Genomic DNA was extracted directly from a peripheral blood sample in EDTA (ethylenediaminetetraacetic acid). The DNA was enriched for the complete coding regions and splice junctions of most genes of the human genome using a proprietary capture system developed by GeneDx (Stamford, CT) for next-generation sequencing with CNV calling (NGS-CNV). The enriched targets were simultaneously sequenced with paired-end reads on an Illumina platform. Bi-directional sequence reads were assembled and aligned to reference sequences based on National Center for Biotechnology Information (NCBI) Reference Sequence (RefSeq) transcripts and human genome build GRCh37/UCSC hg19. Using a custom-developed analysis tool (XomeAnalyzer; GeneDx, Stamford, CT), data were filtered and analyzed to identify sequence variants and most deletions and duplications involving three or more coding exons in the selected genes or regions of interest. Genomic DNA testing of peripheral blood revealed a heterozygous c.5106 G>C p.(Q1702H) (CAG>CAC) in exon 34 of the *ANKRD26* gene.

Notably, the patient had no childhood history of thrombocytopenia. The only hematologic abnormality the patient reported prior to development of AML was hereditary pure red cell aplasia that responded to immunosuppression with ongoing remission since two years of age. The patient experienced relapse of AML after initial induction therapy and there were challenges in finding a donor for hematopoietic stem cell transplant due to her Middle Eastern heritage and lack of HLA-matched family donors. Unfortunately, after developing neutropenic fever for which re-induction therapy was halted, the patient passed away due to complications of fungemia including septic shock and disseminated intravascular coagulation before receiving further cancer-directed treatment.

## Discussion

We present a case of AML in a patient with heterozygous c.5106 G>C p.(Q1702H) (CAG>CAC) in exon 34 of the *ANKRD26* gene, which has not been previously identified at significant frequency in large population cohorts (e.g., gnomAD) nor has it been published as pathogenic or benign. Although the patient had hereditary pure red cell aplasia, she had no known history of platelet disorders or chronic thrombocytopenia prior to AML. The childhood bone marrow evaluation prior to the presence of AML was not available for review. In individuals with active or chronic hematologic neoplasms or conditions, there is a possibility that genomic testing may detect an acquired somatic variant, leading to a false positive result. The clinical sensitivity of the testing performed depends in part on the patient's clinical phenotype and is expected to be highest for individuals with clearly defined disease and/or family history of disease.

*ANKRD26*-related thrombocytopenia is a rare genetic disorder that can increase the risk of developing AML. In a study of 250 consecutive, non-familial, adult AML patients screened for mutations in the first exon of *ANKRD26*, including the 5’UTR, three patients had variants in the *ANKRD26* coding region [5]. Notably, Diamond-Blackfan anemia also increases the risk of AML; however, the initial staging bone marrow biopsy results did not indicate features of Diamond-Blackfan anemia. It remains unclear if the variant of uncertain significance in this patient contributed to development of AML. Given variable penetrance of disease with *ANKRD26* mutations, it is unclear whether cancer risk remains increased in variant phenotypes without prominent childhood thrombocytopenia. Genetic testing was recommended for the patient’s immediate family members, given the unclear significance of this variant. Additionally, genetic evaluation of hematopoietic stem cell transplant donors is essential to avoid transplant from a donor with the same genetic disorder because inadvertent transplant from a donor carrying a germline mutation associated with a hereditary myeloid malignancy syndrome may result in poor engraftment or donor-derived malignancy [6,7].

## Conclusions

*ANKRD26*-related thrombocytopenia is a rare genetic disorder that can increase the risk of developing AML. Genetic testing should be considered in patients with unexplained thrombocytopenia, especially in those with a family history of bleeding or hematologic malignancies. Surveillance for early detection of myeloid neoplasms should include an annual complete blood count with bone marrow examination if abnormalities are noted. Allogeneic stem cell transplantation is a viable treatment option for patients with *ANKRD26*-related AML with appropriate genetic screening of donors prior to transplant. It remains unclear if the c.5106 G>C p.(Q1702H) (CAG>CAC) in exon 34 of the *ANKRD26* gene variant seen in this patient confers an increased risk of malignancy; however, consistent reporting of variants of uncertain significance is essential to accrue patient data on a population level to identify new pathologic variants.

## Figures and Tables

**Figure 1:**
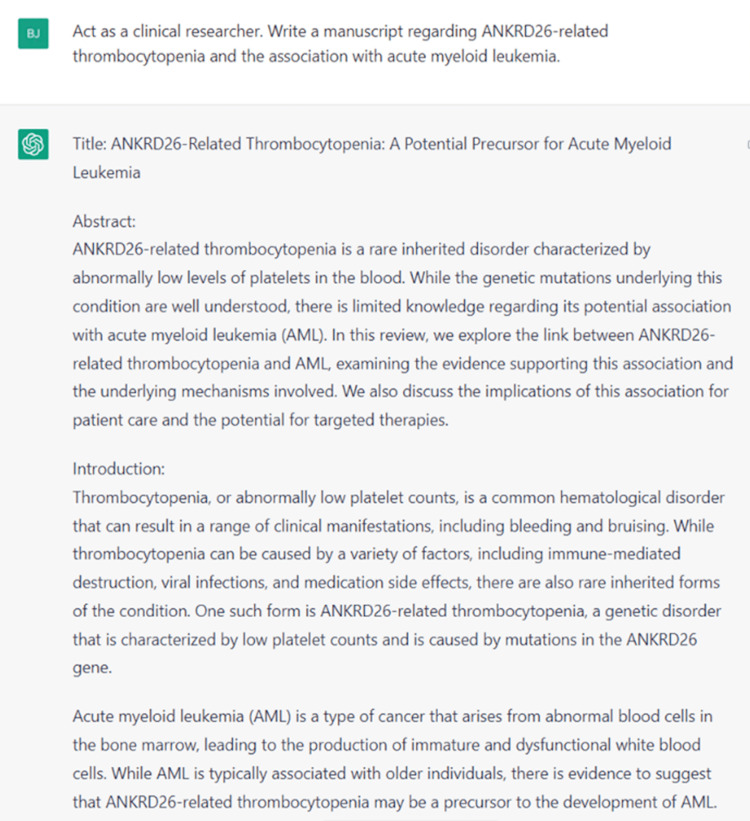
ChatGPT query utilized to write introduction and abstract

**Figure 2:**
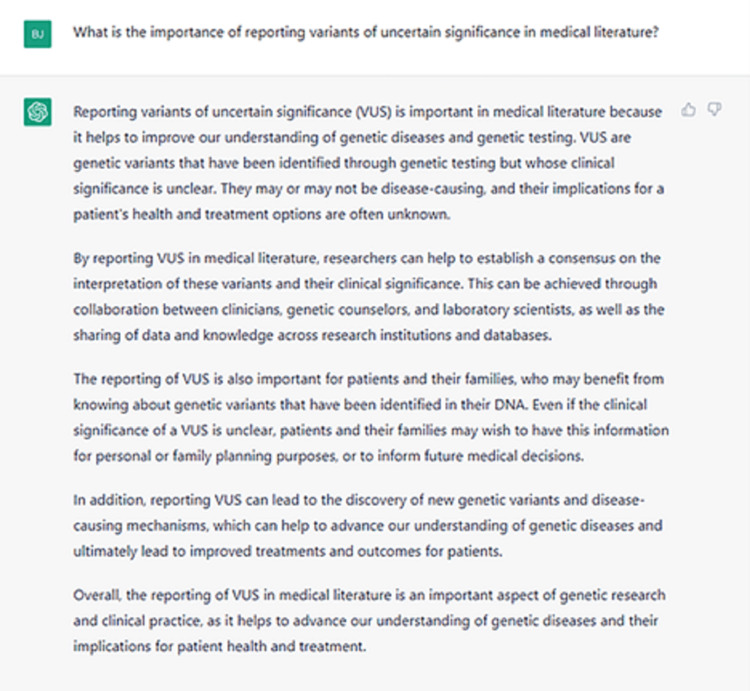
ChatGPT query utilized to emphasize clinical relevance of variants of uncertain significance in conclusion
